# Molecular Pathogenicity of Enteroviruses Causing Neurological Disease

**DOI:** 10.3389/fmicb.2020.00540

**Published:** 2020-04-09

**Authors:** Anna Majer, Alan McGreevy, Timothy F. Booth

**Affiliations:** ^1^Viral Diseases Division, National Microbiology Laboratory, Winnipeg, MB, Canada; ^2^Department of Medical Microbiology and Infectious Diseases, University of Manitoba, Winnipeg, MB, Canada; ^3^Department of Biology, University of Winnipeg, Winnipeg, MB, Canada

**Keywords:** enterovirus, host factor, pathogenesis, central nervous system, neurological disease

## Abstract

Enteroviruses are single-stranded positive-sense RNA viruses that primarily cause self-limiting gastrointestinal or respiratory illness. In some cases, these viruses can invade the central nervous system, causing life-threatening neurological diseases including encephalitis, meningitis and acute flaccid paralysis (AFP). As we near the global eradication of poliovirus, formerly the major cause of AFP, the number of AFP cases have not diminished implying a non-poliovirus etiology. As the number of enteroviruses linked with neurological disease is expanding, of which many had previously little clinical significance, these viruses are becoming increasingly important to public health. Our current understanding of these non-polio enteroviruses is limited, especially with regards to their neurovirulence. Elucidating the molecular pathogenesis of these viruses is paramount for the development of effective therapeutic strategies. This review summarizes the clinical diseases associated with neurotropic enteroviruses and discusses recent advances in the understanding of viral invasion of the central nervous system, cell tropism and molecular pathogenesis as it correlates with host responses.

## Introduction

Enteroviruses (EVs) are single-stranded positive-sense RNA viruses of the family *Picornaviridae* (Ehrenfeld et al., 2010). There are 106 enterovirus types known to infect humans, belonging to the four species *Enterovirus A* through *Enterovirus D.* Polio is caused by three strains within the species *Enterovirus C* and the remaining types are non-polio enteroviruses that includes 21 coxsackievirus A types, 6 coxsackievirus B types, 28 echoviruses and 48 numbered enteroviruses (Simmonds et al., 2020). Three rhinovirus species, *Rhinovirus A* through *Rhinovirus C*, are also classified under the genus *Enterovirus* and include 169 rhinoviruses. Although most EVs cause self-limiting gastrointestinal or respiratory illnesses, a growing number have been found to posses the ability to invade the central nervous system and cause potentially fatal neurological symptoms including encephalitis, meningitis and paralysis. The exact number of EV-associated neurological disease cases remains unknown, but 80% of aseptic meningitis (Morens and Pallansch, 1995) and up to 11% encephalitis cases (Koskiniemi et al., 2001) are speculated to be due to EV infection. Poliovirus is the most widely known EV and is the etiological agent of poliomyelitis that primarily affects infants and children, resulting in lifelong disability or death (Howard, 2005). As we near the global eradication of all 3 poliovirus strains, the incidence of poliomyelitis has plummeted drastically (Jorba et al., 2018). Nevertheless, the emergence of poliomyelitis-like neurological disease called acute flaccid myelitis (AFM) since 2014 clearly indicates a non-poliovirus cause. Recent epidemiological and animal work evidence suggests a strong causal link between AFM cases and EV-D68 outbreaks, a virus which previously had little, if any, clinical significance. As the number of EV species capable of invading the central nervous system and linked to neurological symptoms is growing, these viruses are increasingly being considered as re-emerging pathogens of significant importance to public health.

Our current understanding of these non-polio enteroviruses is limited, especially with regards to their neurovirulence. Without an effective treatment strategy to combat or prevent non-polio EV infections of the central nervous system, better understanding of the neuropathogenic process of neurotropic EVs is highly warranted. Elucidating the molecular pathogenesis of these viruses is paramount for the development of effective therapeutic strategies. This review summarizes clinical diseases associated with some of the most common neurotropic enteroviruses and discusses recent understanding of viral invasion into the central nervous system, cell tropism and molecular pathogenesis as it correlates with host responses during neurotropic enterovirus infections.

## Neurological Manifestation of Enterovirus Infections

Numerous EVs are linked to debilitating and potentially deadly neurological diseases including aseptic meningitis, encephalitis and AFM. In certain instances, EV infections are associated with the development of neurological sequelae years after the onset of acute disease, as is suspected for post-polio syndrome (Ramlow et al., 1992) and Guillain-Barré syndrome (Ooi et al., 2010). Here we will briefly describe these disorders and highlight which non-polio EVs are primarily associated with these neuropathies (Table 1).

**TABLE 1:** Enteroviruses associated with neurological illness.

Neurological Disease	Enterovirus	Reference
Acute flaccid myelitis	**EV-A71**	Chonmaitree et al., 1981; Lin et al., 2003
	**EV-D68**	Kreuter et al., 2011; Yea et al., 2017; Dyda et al., 2018
	EV-D70	Bharucha et al., 1972
	EV-B93	Junttila et al., 2007
	EV-D94	Junttila et al., 2007
	Echovirus 33	Grimwood et al., 2003
Encephalitis	**CVA2**	Chiang et al., 2019
	**CVA9**	Zhang et al., 2013
	CVB1	Zhang et al., 2013
	**CVB4**	Geller and Condie, 1995; Zhang et al., 2013
	**CVB5**	Ramelli et al., 2004; Papa et al., 2006
	**Echovirus 4**	McIntyre and Keen, 1993; Logotheti et al., 2009; Zhang et al., 2013
	**Echovirus 5**	Dumaidi et al., 2006
	**Echovirus 6**	Siafakas et al., 2004; Zhang et al., 2013
	**Echovirus 9**	Dalwai et al., 2009; Zhang et al., 2013
	**Echovirus 11**	Moline et al., 2018; Lopez et al., 2019
	Echovirus 17	Hart and Miller, 1973
	Echovirus 19	Kumar et al., 2011; Singh et al., 2016
	Echovirus 21	Singh et al., 2016
	**Echovirus 30**	Siafakas et al., 2004; Zhang et al., 2013
	**EV-A71**	Chonmaitree et al., 1981; Lin et al., 2003
	**EV-D68**	Kreuter et al., 2011; Yea et al., 2017; Dyda et al., 2018
	EV-B75	Avellón et al., 2006; Lewthwaite et al., 2010
	EV-A76	Pallansch et al., 2013
	EV-A89	Pallansch et al., 2013
Meningitis	**CVA2**	Chiang et al., 2019
	**CVA9**	Zhang et al., 2013
	CVA16	Goto et al., 2009
	CVA5	Pallansch et al., 2013
	CVA7	Ranzenhofer et al., 1958; Yamayoshi et al., 2012
	**CVB4**	Geller and Condie, 1995; Zhang et al., 2013
	**CVB5**	Ramelli et al., 2004; Papa et al., 2006
	**Echovirus 4**	McIntyre and Keen, 1993; Logotheti et al., 2009; Zhang et al., 2013
	**Echovirus 5**	Dumaidi et al., 2006
	**Echovirus 6**	Siafakas et al., 2004; Zhang et al., 2013
	**Echovirus 9**	Dalwai et al., 2009; Zhang et al., 2013
	**Echovirus 11**	Moline et al., 2018; Lopez et al., 2019
	Echovirus 14	Chen et al., 2017
	Echovirus 16	Baron et al., 1982
	Echovirus 25	Zhang et al., 2013
	**Echovirus 30**	Siafakas et al., 2004; Zhang et al., 2013
	Echovirus 31	Kelen et al., 1964
	**EV-A71**	Chonmaitree et al., 1981; Lin et al., 2003
	**EV-D68**	Kreuter et al., 2011; Yea et al., 2017; Dyda et al., 2018
Poliomyelitis	Poliovirus	Horstmann, 1949

*Bolded enteroviruses have been linked to cause multiple neurological diseases.*

### Enterovirus-Associated Meningitis and Encephalitis

Enterovirus infections are the most common cause of both meningitis and encephalitis in children 17 years of age and younger (Hasbun et al., 2019). Meningitis represents inflammation of the membrane lining the brain and spinal cord, known as meninges while encephalitis depicts inflammation of the brain parenchyma. Infants are highly susceptible, experiencing mortality rates as high as 10% (Rhoades et al., 2011). Diagnosis is made by several factors including clinical symptoms, neuroimaging and lumbar puncture to assess the cerebral spinal fluid (CSF) for infectious agents (Venkatesan et al., 2013). Enterovirus-specific PCR of CSF specimens is highly recommended although is insufficient for diagnosis when used alone (Venkatesan et al., 2013) due to low yield of positive results (Pérez-Vélez et al., 2007; Lopez et al., 2019). Testing additional sites (i.e., throat and gastrointestinal tract) where virus shedding is prolonged (Han et al., 2010) is recommended but may still result in EV negative status if sample collection is delayed. It is therefore possible that the etiology of meningitis and encephalitis cases due to EV infection are much higher than reported.

Numerous EVs have been detected in cases of encephalitis, aseptic meningitis or meningoencephalitis – inflammation of both meninges and the brain. The wide diversity of enteroviruses associated with encephalitis or meningitis highlights their collective neuropathogenic potential and speaks to their unique tropism. Specifically, poliovirus, CV (A2, A9, B5), echoviruses (6, 9, 11, 30) and EV-A71 are commonly implicated in causing both encephalitis and aseptic meningitis (Jain et al., 2014; Pons-Salort et al., 2015; Rudolph et al., 2016). However, EV-A71 was also linked to cause outbreaks of brainstem encephalitis or AFM (Lee, 2016). CV (A5, A7, A16, B2, B3, B4) and echoviruses (14, 16, 25, 31) are all implicated in cases of meningitis, while CV (A2, B1), echoviruses (4, 5, 17, 19, 21) and EV (75, 76, 89) are implicated in cases of encephalitis (Jain et al., 2014; Pons-Salort et al., 2015; Rudolph et al., 2016).

### Acute Flaccid Paralysis and Acute Flaccid Myelitis

Acute flaccid paralysis (AFP) is a WHO reportable disease in children under the age of 15. AFP is clinically defined as acute onset of flaccid paralysis of one or more limbs due to an infectious cause (The United Kingdom Acute Flaccid Paralysis AFP Task Force, 2019). The disease may damage different parts of the body including spinal cord, peripheral nerves, neuromuscular junctions and muscles. Poliovirus was the primary etiology of infectious AFP cases causing poliomyelitis. With current near-universal vaccination strategies designed to eradicate poliovirus, the total number of AFP cases attributed to poliovirus around the world have decreased drastically (Suresh et al., 2018).

A subset of AFP called acute flaccid myelitis (AFM) represents a disease where paralysis and limb weakness typically occurs within a week of respiratory symptoms or fever caused by a non-polio viral infection. In addition to the acute limb weakness, a lesion in the spinal cord gray matter that spans at least one vertebra and often elevated leukocyte counts within the CSF is a classic diagnostic feature (Dyda et al., 2018; Centers for Disease Control and Prevention,, 2019; Fatemi and Chakraborty, 2019). Symptoms result from inflammation followed by the loss of motor neurons within the brain stem and spinal cord without any signs of generalized encephalitis (Maloney et al., 2015; Hovden and Pfeiffer, 2015). AFM cases have been associated with several viral pathogens including enteroviruses and echoviruses (Gilsdorf, 2019). EV-D68 is the most consistent enterovirus linked with AFM (Kreuter et al., 2011), accounting for up to 50% of all confirmed AFM cases (Lopez et al., 2019), but other enteroviruses such as EV-A71 (Chumakov et al., 1979; Lopez et al., 2019; Lee et al., 2014), CVs (Cho et al., 2017; Lopez et al., 2019), EV-D70 (Bharucha et al., 1972), EV-B93 and EV-D94 (Junttila et al., 2007), echovirus 33 (Grimwood et al., 2003) and echovirus 11 (Moline et al., 2018; Lopez et al., 2019) have also been identified but at a much lower frequency. Although AFM is rare, affecting more than 500 confirmed cases worldwide since 2012 (Helfferich et al., 2019), the prognosis is poor with less than 20% of children fully regaining neurological function within 6 months (Sejvar et al., 2016; Yea et al., 2017; Helfferich et al., 2019). No treatment strategy currently exists for AFM patients but physical rehabilitation which, when implemented during the acute phase of the illness, slightly helps improve long-term neurological outcomes (Van Haren et al., 2015). Latest nerve transfer procedures also show promise in restoring partial function to paralyzed limbs in AFM patients (Pino et al., 2019).

Recent epidemiological criteria using the Bradford-Hill analyses showed strong support that AFM is primarily caused by EV-D68 (Dyda et al., 2018; Messacar et al., 2018). The particular cyclical and biennial patterns of EV-D68 infections correlated well with outbreaks of AFM in children occurring in 2014, 2016, and 2018 (Dyda et al., 2018; Kramer et al., 2018). However, detecting the virus from clinical samples remains a challenge and contributed to skepticism surrounding EV-D68 as a cause of AFM. During 2018, the active surveillance network in the USA confirmed approximately 230 AFM cases of which approximately 44% of respiratory, 13% of stool and 3% of CSF specimens were EV-D68 positive (Lopez et al., 2019). Recent animal models that mimicked human disease and satisfied Koch’s postulates helped solidify the link between EV-D68 infection and subsequent AFM complications. Intraperitoneal inoculation of EV-D68 into neonatal mice recapitulated clinical AFM symptoms including paralysis due to motor neuron loss in the anterior horn cells of the spinal cord (Hixon et al., 2017). Virus isolated from spinal cord lysates of paralyzed mice resulted in cell death in cultured cells and caused paralysis when injected into mice (Hixon et al., 2017). Similarly, intraperitoneal infection of EV-D68 into neonatal BALC/c mice induced both interstitial pneumonia and AFM (Sun et al., 2019). Studies within these types of animal models will help unravel the molecular mechanisms used by EV-D68 to casue AFM.

### Chronic Neurological Diseases Associated With Enterovirus Infections

Some poliovirus infected patients develop a condition called post-polio syndrome (PPS) that is characterized by muscle weakness and atrophy several decades following acute infection but, in contrast to poliomyelitis, these sequelae tend to be transient and progressive (Huang and Shih, 2015). It is commonly a diagnosis of exclusion once other possible medical or surgical causes of gradual onset weakness are discounted, occurring on average 30–35 years after acute poliomyelitis (Boyer et al., 2010). The disease is more common in patients who had severe initial poliovirus infections and subsequently developed permanent impairment (Ramlow et al., 1992). Studies vary widely in the estimated prevalence of PPS, ranging from 31% (Ragonese et al., 2005) to 85% (Takemura et al., 2004) of polio infected individuals. A case reported in 2017 described a post-polio-like symptom several decades following a severe infection with EV-70, suggesting that polio may not be the only enterovirus capable of inducing this syndrome (Suzuki et al., 2017).

The precise mechanism of PPS is unknown and treatment is limited to supportive measures (Lin and Lim, 2005). Muscle weakness in PPS is asymmetrical and is more likely, but not exclusive to muscles that were originally affected by poliomyelitis (Lin and Lim, 2005). One proposed mechanism of PPS stems from the properties that peripheral neurons are capable of axonal regrowth and/or sprouting. For muscle weakness to be clinically apparent, more than 50% of spinal anterior horn neurons that innervate the muscle must be lost (Lin and Lim, 2005). Denervation of muscle can stimulate terminal axons of surviving neighboring neurons to sprout, re-innervating muscle fibers that lost neuronal connection with the spinal cord (Boyer et al., 2010). As denervation can be caused by neuronal cell death, this proposed mechanism helps explain the restoration of function following acute poliomyelitis in PPS patients (Boyer et al., 2010). In support of this proposed mechanism, the rate of residual disability following poliomyelitis was lower in patients with PPS (Klingman et al., 1988). However, the sprouting re-innervation of terminal axons does not appear to be stable and intense muscle use may accelerate deterioration of the terminal axon sprouts (Boyer et al., 2010). Therefore, the benefits of strength training exercises in PPS patients remains controversial where some find benefits (Chan et al., 2003) while others suggest that muscle exhaustion leads to accelerated weakness (Pastuszak et al., 2017).

Guillain-Barré syndrome (GBS) is an inflammatory immune disorder characterized by rapid-onset muscle weakness due to damage to the peripheral nervous system (PNS). The initial symptoms of GBS are often changes in sensation or pain in the extremities that develop over hours to weeks. In some cases, weakness of breathing muscles during the acute phase of the syndrome requires mechanical ventilation but, if the patient survives this phase, complete recovery is likely (Gear, 1984). Despite being classified as a single syndrome, GBS appears to result from one of two related but distinct etiologies: (1) a viral infection of CNS tissue, possibly of Schwann cells, that induces inflammation of the myelin sheaths and leads to their degradation; or (2) an autoimmune reaction to the myelin sheaths initiated in response to an infection elsewhere in the body, vaccination or drug reaction (Gear, 1984). The distinction between these two etiologies is largely made on patient history and whether virus can be detected from the CSF. Recent cases of AFM caused by EV-D68 can be distinguished from GBS via MRI, as GBS tends to be systemic and descending while AFM more commonly presents as asymmetric weakness (Gill et al., 2018). However, the overlapping clinical symptoms of muscle weakness and myelitis make it entirely possible that some GBS cases are due to enterovirus infections in which the virus is not successfully isolated, or that enterovirus infections may precipitate a GBS attack (Pallansch et al., 2013). In fact, EV-A71 outbreaks have linked the virus to GBS (Ooi et al., 2010) and one case study identified GBS with peripheral nerve demyelination following hand foot and mouth disease (HFMD), though the specific enterovirus that caused the HFMD was not identified (Mori et al., 2000). It still remains largely unclear how often and in what capacity enteroviruses contribute to the development of GBS.

## Routes of Enterovirus Neuroinvasion

Viral pathogens employ a variety of strategies to gain entry into the CNS (Kim, 2008) and uncovering these routes of neuroinvasion could reveal potential avenues that can be targeted to prevent EV-induced neurological disease. EVs replicate in either the gastrointestinal tract (i.e., poliovirus, most numbered enteroviruses and echoviruses) or the lungs (i.e., EV-D68) during early disease. Poliovirus is an excellent example of a gastrointestinal enterovirus which uses retrograde axonal transport within motor neurons to enter the CNS. Poliovirus primarily infects gastrointestinal epithelia by binding to poliovirus-specific receptor CD155, and thereby gains entry into the lymphatic and circulatory systems (reviewed in Gilsdorf, 2019). The virus then spreads within the circulatory system (i.e., viremia) and disseminates to infect peripheral tissues such as muscles. At neuromuscular junctions, poliovirus enters motor neurons by using receptor-mediated endocytosis allowing the virus to travel from the terminal to the cell body within the endosome by retrograde axonal transport (Racaniello and Ren, 1994; Gromeier and Wimmer, 1998; Mueller et al., 2002; Ohka et al., 2004). Physiologically, the virus moves from the muscle to the sciatic nerve, enters the spinal cord and eventually reaches the brain (Ren and Racaniello, 1992; Ohka et al., 1998). In support, surgically severing nerves in mice prior to poliovirus infection prevented the spread of the virus to the spinal cord (Gromeier and Wimmer, 1998). Interestingly, retrograde axonal transport of poliovirus is fairly inefficient, which could explain the low incidence of neurological complications seen in patients (Lancaster and Pfeiffer, 2010). However, local muscle injury significantly enhanced poliovirus neuroinvasiveness, allowing for 3-fold more virus to enter the CNS (Lancaster and Pfeiffer, 2010). Retrograde axonal transport was demonstrated for several non-polio enterovirus such as EV-A71 (Chen C.S. et al., 2007) and EV-D68 (Hixon et al., 2019a).

The ability of enteroviruses to infect immune cells is another potential mechanism for their neuroinvasion. Numerous EVs were found to infect circulating immune cells which can serve as a Trojan Horse to deliver the virus into the CNS tissue. For example, the myeloid-like Mac3^+^ peripheral blood mononuclear cells (PBMCs) were found to be highly susceptible to CVB3 infection (Tabor-Godwin et al., 2010). In a neonatal mouse model these cells were recruited to the CNS via the choroid plexus, allowing for the virus to gain unrestricted entry into the brain (Tabor-Godwin et al., 2010). Infection of mice by CVB3 showed that B cells were susceptible to viral infection and also helped disseminate the virus during early infection to the brain and other tissues throughout the body (Mena et al., 1999). CVB3 was shown to replicate in several *in vitro* cell lines including Raji (B cell), Jurkat (T cell) and U-937 (monocyte) (Hwang et al., 2012) implicating these cell types to possibly serve as viral shuttles into the CNS. Poliovirus was shown to infect monocytes (Freistadt et al., 1993; Freistadt and Eberle, 1996), EV71 was able to replicate in CD14+ cells (Wang J. et al., 2013), dendritic cells (Lin et al., 2009) and PBMCs (Wongsa et al., 2019) while echoviruses (1, 7, 8, and 9) replicated in mature dendritic cells isolated from PBMCs but not monocytes (Kramer et al., 2007). Although a wide range of circulating immune cells are susceptible to diverse enteroviruses, further studies are needed to assess the extent of viral invasion into the CNS by utilizing immune cells as shuttles.

Another possible mechanism of neuroinvasion is through the direct infection of natural barriers that encase the brain and spinal cord. The blood-brain barrier (BBB) functions to restrict entry of large molecules as well as cells and pathogens into the brain from the circulatory system. Similarly, the choroid plexus found in each of the four ventricles of the brain controls the production of CSF and serves as the blood-cerebrospinal fluid barrier (BCSFB), preventing passage of most pathogens into the brain while permitting the delivery of nutrients and removal of metabolic wastes. The BCSFB is a site of CNS immune surveillance, providing a more permissive barrier for immune cells to traverse, while the BBB is more resistant to the movement of both host cells and invasive pathogens (Ransohoff and Engelhardt, 2012). Some pathogens appear to be limited to crossing only the BBB, while others are able to traverse both the BBB and BCSFB by using various mechanisms (Dahm et al., 2016). Both CVB3 (Puccini et al., 2014) and echovirus 30 (Schneider et al., 2012) are able to directly infect the BCSFB. Intracranial infection of CVB3 into newborn mice revealed the presence of viral RNA in the choroid plexus (Feuer et al., 2003). Similarly, echovirus 30 was found to directly infect BCSFB cells from both the apical and basolateral membranes without compromising barrier integrity as demonstrated using human choroid plexus papilloma cells *in vitro* (Schneider et al., 2012). However, echovirus 30 infection of the barrier did not stimulate T cell migration into the CSF which is typically observed during enteroviral meningitis (Lucht et al., 1992) indicating that other factors are required to initiate this immune response. In turn, poliovirus can infect human brain microvascular endothelial cells, an *in vitro* model of the BBB, which would permit the shed of progeny virus into the brain and/or damage the barrier by lysing the endothelia (Coyne et al., 2007). Overall, these studies demonstrate that once EV virions are found within the blood they can induce lytic and non-lytic mechanisms by which to cross these barriers to reach the CNS.

One important factor in CNS invasion is the generation of genetic variants, otherwise known as quasispecies, which are produced during viral replication (Rhoades et al., 2011). Enteroviruses have a relatively low fidelity RNA-dependent RNA polymerase compared to eukaryotic hosts or DNA viruses (Ward and Flanegan, 1992) and high-fidelity polymerase has been correlated with decreased poliovirus fitness under selective pressure (Pfeiffer and Kirkegaard, 2005). The production of viral quasispecies was shown to be an important component for poliovirus neuroinvasion. This was demonstrated by infecting mice with a poliovirus isolate (G64S) containing a high-fidelity polymerase which made the isolate less neuroinvasive than its wild-type counterpart (Vignuzzi et al., 2006). The neurotropism and pathogenesis of the G64S poliovirus isolate was restored when chemical mutagenesis was employed to create quasispecies before inoculating into the animal model (Vignuzzi et al., 2006). This data showed that genetic diversity produced during enterovirus replication seems to induce neuroinvasive capabilities. However, selective pressures within the host plus potential bottlenecks naturally restrict viral spread throughout the body. To identify potential bottlenecks, four restriction-enzyme tagged poliovirus strains of equivalent fitness were inoculated into mice. The authors found only a subset of these strains within mouse brain, suggesting the existence of a bottleneck between the site of inoculation and brain tissue (Pfeiffer and Kirkegaard, 2006). This bottleneck was overcome by inoculating with high viral titers, indicating that the bottleneck is not a physical barrier but an immunological one. The authors suggested that once the founder virus reached the brain an antiviral state was initiated resulting in a “burned-bridge” phenomenon that limited subsequent virus strains from entering the CNS (Pfeiffer and Kirkegaard, 2006). This phenomenon was recently observed in clinical isolates of EV-A71 infected specimens where diverse number of quasispecies was detected in the respiratory and digestive samples while a dominant haplotype mutation in the VP1 region emerged in isolates collected from the CNS (Huang et al., 2017). This haplotype conferred enhanced growth and fitness in human neuronal cells (Huang et al., 2017). Further characterization of quasispecies generated at different biological sites during mild and severe cases of neurological disease would be valuable to identify the EV haplotypes that confers neuroinvasion.

## Enterovirus Tropism Within the Nervous System

Clinical observations and animal models have revealed lesions within the CNS that indicate unique tissue tropisms for different enteroviruses. Poliovirus primarily affects the anterior horns of gray matter in the spinal cord, which are composed of motor neurons innervating skeletal muscle (Jubelt et al., 1980; Brown et al., 1987). Poliovirus receptors are highly expressed in synaptosomes and the neuromuscular junctions provide the most accessible sites outside the CNS for poliovirus binding (Brown et al., 1987). Similarly, EV-D68 damages motor neurons of the anterior horn in the spinal cord and brain stem leading to lesions visible on the MRI within these structures (Knoester et al., 2019). EV-D68-induced paralytic myelitis animal model further revealed that virus was present within motor neurons of the anterior horn of the spinal cord segments that corresponded to the paralyzed limbs (Hixon et al., 2017). In contrast, EV-A71 is associated with extensive lesions that were previously detected within the brainstem, pons, medulla, cerebellum, cortex, thalamus, dentate nuclei and cerebrum (Shieh et al., 2001; Nagata et al., 2002; Kao et al., 2004; Yu et al., 2014). The reason for such diverse tropism by EV-A71 remains largely unknown. However, encephalitis of the midbrain, pons and medulla occurs in 62% of patients with neurological complications during EV-A71 infection (Wang et al., 1999) and, due to the role of the brainstem in autonomic regulation, encephalitis of this anatomical region is typically associated with pulmonary edema and fluctuating blood pressure (Liao et al., 2015). In terms of CVs, a mouse model designed to imitate neonatal CVB3 infections found lesions in the hippocampus and cortex, although viral RNA was detected throughout the brain (Wang et al., 2014). The choroid plexus and subventricular zone, a site of neurogenesis, were found to contain CVB3 viral proteins in another animal model; a pathology that is further supported by *in vitro* studies showing susceptibility of neural progenitor cells to CVB3 (Puccini et al., 2014). Infection of these neuroprogenitor cells may represent developmental abnormalities seen as a result of CV infection (Feuer et al., 2005). A neonatal mouse model of CVB5 revealed the presence of viral antigen and necrosis of neurons within the cerebral cortex and the entire spinal cord in addition to necrosis of hindlimb muscles and cardiomyocytes (Mao et al., 2018). These studies highlight the diverse neurotropism exhibited by enteroviruses which help explain some of the neurological symptoms.

## CNS Cell Types Susceptible to Enteroviruses

The interplay between EV infected cells and host response is crucial to understand the progression of neurological disease. As each cell type within the CNS would invoke a slightly different response upon infection, identifying which cell type is susceptible to which EV species will provide additional clues as to the progression of CNS disease development. To date, numerous studies using animal models and *in vitro* cultures not only confirmed the neurotropic ability of numerous enteroviruses but also revealed that these viruses readily infect neuronal progenitors, mature neurons and glia cells such as astrocytes. For example, both EV-A71 and CVB3 were detected in undifferentiated neuronal progenitor cells after intraperitoneal inoculation of virus into a neonatal animal model (Huang et al., 2014; Puccini et al., 2014). In tissue culture models, EV-A71 and CVA16 infected human mature neuroblastoma cell line (SK-N-SH) and caused necrosis (Yogarajah et al., 2017b: Yu et al., 2017). Similarly, neurotropic EV-D68 strains post 2014 outbreak were able to infect and replicate in the human neuroblastoma cell line (SH-SY5Y) and human postnatal cortical neuron cultures as compared to the non-neurotropic strains (Brown et al., 2018). The authors found that only a single round of viral replication occurred from transfecting pre-2014 outbreak viral RNA into neuroblastoma cells suggesting that viral entry into the cell was the primary neurotropic factor (Brown et al., 2018). In support of this observation, a recent study using chimeras revealed that cellular and tissue tropism of EVs as well as acid sensitivity is dependent on the viral capsid protein (Royston et al., 2018). Specifically, the authors used EV-D68 (respiratory isolate) that was unable to replicate in human neuroblastoma cells (SH-SY5Y) or neuronal tissues and EV-D94 (gastrointestinal isolate) which was able to replicate in both models to high titers. When the authors generated an enteroviral chimera that expressed the capsid protein of EV-D68 and the rest of the virus was derived from EV-D94, the ability to infect neuronal cells and tissues was abrogated and the chimeric virus exhibited the same acid sensitivity and cellular tropism as EV-D68 (Royston et al., 2018). It is therefore not surprising that primary mouse hippocampal neurons expressing the human CD155 receptor were susceptible to poliovirus infection (Daley et al., 2005). A recent study found that EV-D68 strains from pre and post 2014 outbreak infected and replicated in spinal motor neurons differentiated from human-derived induced-pluripotent stem cells (iPSC) (Hixon et al., 2019a). Interestingly, infection of motor neuron-like mouse cells (NSC-34) by EV-A71 produced a strain-dependent, non-lytic infection that released viable viral particles from the cell via autophagy (Too et al., 2016). These studies reinforce the diverse neurological manifestation observed in EV infections.

Additional host factors are likely important for mediating neuropathogenicity based on a recent study showing that not all post-2014 outbreak EV strains caused neurological disease in mice (Hixon et al., 2017). This premise is further supported by recent work using mouse organotypic brain slice cultures. Both pre- and post-2014 outbreak EV-D68 strains were able to infect and replicate within these cultures, suggesting that the neurotropic potential was not a recently acquired phenotype (Rosenfeld et al., 2019). Instead, the authors propose that the immune response is responsible for modulating neuroinvasive properties of EV-D68 (Rosenfeld et al., 2019). Recently, EV-A71 infected adult mice containing a humanized immune system where not only susceptible to a clinical isolate of EV-A71 but recapitulated clinical symptoms and histopathology of disease such that the viral antigen was detected throughout the spinal cord and several regions within the CNS (Ke et al., 2019). As wild-type mice were resistant to EV-A71 infection, this study further reinforces the critical role the immune response plays in establishing neurological disease.

Astrocytes are the most abundant cell type within the human brain (Volterra and Meldolesi, 2005) and perform numerous diverse roles. These cells support the function of endothelial cells that make up the BBB, provide nutrients and metabolize neurotransmitters for use by neurons and play a role in CNS repair after injury (Pascual et al., 2005). In terms of EV infections, tissue culture models of astrocytes are permissive to numerous enterovirus strains including EV-A71, CVA9, CVB3, CVB4 and EV-D68 (Kwon et al., 2004; Tung et al., 2010; Haolong et al., 2013; Zeng et al., 2013; Du et al., 2014; Wang C. et al., 2015; Rosenfeld et al., 2019). In fatal cases of EV-A71, the viral antigen was detected by histology within neurons and astrocytes (Yan et al., 2000; Yu et al., 2014). Similarly, post-mortem brain tissues from patients with confirmed EV-A71 infection and brain tissue from a non-human primate model of EV-A71 revealed that more than 80% of EV-A71 antigen was detected in astrocytes (Feng et al., 2016). As EV-D68 infected hiPSC derived astrocytes produced 2x more virus within 24 h post infection than 3 days post infection in neurons (Rosenfeld et al., 2019), it suggests that glia may be important for rapid viral propagation within the CNS. Further *in vitro* functional studies identified that EV-A71 infected astrocyte cultures released pro-inflammatory cytokine IL-6 which increased secretion of excitatory neurotransmitters in bystander neurons that could have profound consequences on neuronal function (Feng et al., 2016) and immune response within the CNS. Collectively, this data highlights the important role astrocytes play in the development of neurological complications observed during EV infection. Additional studies are highly warranted to assess the impact of enterovirus-infected glial cells on neurological disease.

## Receptors for Neurotropic Enteroviruses

One important determinant of viral tropism is the expression of the viral-specific receptor(s) on the cell surface. Ever since CD155 was first identified in 1989 as the poliovirus receptor (Mendelsohn et al., 1989), the extracellular receptors for many different enteroviruses have been uncovered. These studies made clear that enteroviruses can use a wide array of receptors and attachment factors for cell entry (Table 2). For example, EV-A71 is known to bind to 2 receptors and several potential attachment factors, which reflects its ability to infect different cell types. Scavenger receptor B2 (SCARB2) (Yamayoshi et al., 2009) is expressed in human neurons and glial cells (Jiao et al., 2014) and EV-A71 infection of a transgenic mouse model expressing human SCARB2 caused ataxia, paralysis and death of the animals (Fujii et al., 2013). Another EV-A71 receptor called human P-selectin glycoprotein ligand-1 (PSGL-1) is expressed primarily on leukocytes and is bound by select EV-A71 strains (Nishimura et al., 2009). Additional attachment molecules that enhance viral infectivity and contribute to viral dissemination and neurotropism include heparan sulfate glycosaminoglycans (HS) (Tan et al., 2013), sialic acid (Yang et al., 2009), annexin II (Yang et al., 2011), nucleolin (Su et al., 2015), vimentin (Du et al., 2014) and heat shock protein 70 (Xu et al., 2019) among others (reviewed in Owino and Chu, 2019). Other receptors utilized by enteroviruses for cell entry that may contribute to neuropathogenicity include the decay-accelerating factor (DAF or CD55, part of the complement cascade) used by CVA21, echovirus 6 and echovirus 11 (Shafren et al., 1997; Lea et al., 1998; Renois et al., 2011) or Coxsackievirus and Adenovirus Receptor (CAR), which is the main entry receptor for Coxsackie B viruses (Martino et al., 2000). Significantly, CD55 is expressed on neurons within the gastrointestinal system and glia within the CNS tissue (Gelderman et al., 2004), while CAR is abundantly expressed in the brain, with highest expression levels observed in newborn mice (Honda et al., 2000). Specifically within the motor neuron cell line NSC-34, infection by EV-A71 was found to rely on the surface expressed Prohibitin (PHB), suggesting that the virus uses this protein as a receptor for entry into motor neurons (Too et al., 2018). Additional studies are necessary to determine if this is a cell type and viral strain specific receptor. In turn, EV-D68 was primarily found to depend on sialic acid for entry which allowed subsequent viral uncoating (Liu et al., 2015) and facilitated genome release into the cytoplasm. Sialic acid-mediated viral entry was also documented for other enteroviruses such as EV-D70 (Alexander and Dimock, 2002; Nokhbeh et al., 2005), EV-A71 (Yang et al., 2009; Su et al., 2012) and CVA24 (Nilsson et al., 2008). Recent neuronal-specific studies revealed that cleavage of the sialic acid receptor prevented infection of human motor neurons derived from iPSCs by only the pre-2014 outbreak EV-D68 strains while post-2014 strains were unimpeded, suggesting that contemporary EV-D68 strains used another neuronal-specific receptor for cell entry (Hixon et al., 2019a).

**TABLE 2:** Receptors used by neurotropic enteroviruses for cell entry.

Virus	Receptor	Reference
CVA16	PSGL-1; SCARB2	Yamayoshi et al., 2009, 2012; Nishimura and Shimizu, 2012
CVA21	CD55; ICAM-1	Shafren et al., 1997
CVA7	SCARB2	Yamayoshi et al., 2012
CVA9	αVβ3, αVβ6 integrins	Harvala et al., 2003
CVB1 to CVB6	CAR	Bergelson et al., 1997; Carson et al., 1997
CVB1 and CVB5	CD55	Shafren et al., 1995
Echovirus 5	Heparan sulfate; FcRn	Israelsson et al., 2010; Morosky et al., 2019
Echovirus 6	CD55; Haparan sulfate; FcRn	Goodfellow et al., 2001; Renois et al., 2011; Morosky et al., 2019
Echovirus 9	αVβ3 integrin, FcRN	Ylipaasto et al., 2010; Morosky et al., 2019
Echovirus 11	CD55; FcRn	Lea et al., 1998
EV-A71	PSGL-1; SCARB2; sialic acid	Yamayoshi et al., 2009; Su et al., 2012
EV-D68	ICAM-5	Wei et al., 2016
Poliovirus	CD155	Mendelsohn et al., 1989; He et al., 2000

The intracellular adhesion molecule 5 (ICAM-5) was identified to facilitate infection by contemporary EV-D68 in otherwise non-permissive Vero cells following a sialic-acid independent mechanism (Wei et al., 2016). Although this receptor is abundantly expressed in neurons (Gahmberg et al., 2008) and shows promise for neuronal-specific tropism of neurotropic enteroviruses, the pattern of EV-D68 infection in brain slice models and microfluidic chamber motor neuron cultures does not correspond to the distribution or expression of ICAM-5 (Hixon et al., 2019a). In fact, ICAM-5 expression in human iPSC-derived motor neuron-like cells was observed within the soma and dendrites while EV-D68 viral particles were observed on the axon terminals (Hixon et al., 2019b). Furthermore, ICAM-5 was not detected in neonatal mouse spinal cords where EV-D68 is preferentially found (Hixon et al., 2019b). It is possible that a homolog to ICAM-5 is the true neuronal-specific receptor for EV-D68 (Hixon et al., 2019b).

An important factor to consider in neuropathogenicity is that EVs can gain the use of additional receptors through adaptation during replication. Receptor adaptation has been well documented for numerous enteroviruses (reviewed in Cagno et al., 2019), highlighting their inherent potential for invading the CNS as a result of adaptation. For example, a non-synonymous single amino acid change within the VP1 region of EV-A71 was identified in isolates obtained from the blood and CSF samples of an immunocompromised host as compared to respiratory specimens (Cordey et al., 2012). This mutation improved viral growth in neuroblastoma cells (SH-SY5Y) (Cordey et al., 2012; Tseligka et al., 2018) due to the virus gaining the ability to bind to HS on the cell surface (Tseligka et al., 2018). Similarly, EV-D68 infection of RD cells resulted in mutations located within the VP1 and VP2 regions that allowed the virus to not only bind the sialic acid receptor but also sulfated glycosaminoglycans for entry (Baggen et al., 2019). Despite our knowledge of these receptors and attachment factors, it is still unclear why neurotropic EVs localize more commonly to certain regions of the CNS (Lee, 2016).

## Cellular Mechanisms of Enterovirus Neuropathogenicity

The extensive phenotypic diversity of enteroviruses has prevented the development of a universal vaccine or therapeutic to combat these diseases. Identification of possible common cellular mechanisms that are involved in EV infection, specifically within the nervous system, would provide unprecedented strides into devising a potential pantropic antiviral therapeutic strategy. In several studies, EV replication within neurons reached lower viral titers over a longer incubation period than in non-neuronal cells, implicating intrinsic cell-specific host factors that contribute to viral pathogenicity (Daley et al., 2005; Yogarajah et al., 2017b). Here we will briefly highlight some of the main host-virus interactions identified for neurotropic enteroviruses and their potential impact on neuropathogenicity. Examples of host factors involved in viral entry and replication, immune response and cell death will be further discussed.

### Host Factors Involved in Enterovirus Entry and Replication

**FIGURE 1:**
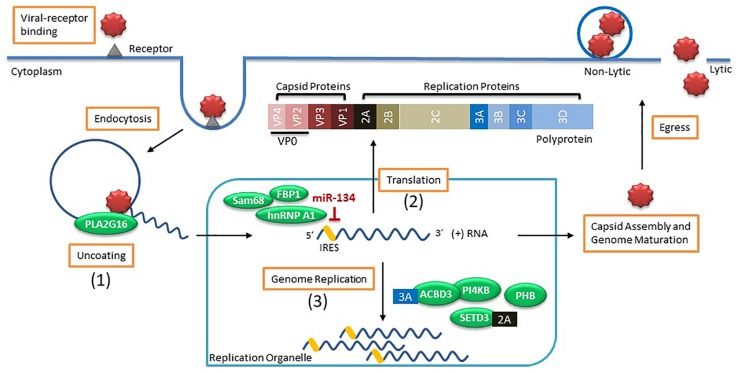
Enterovirus life cycle and the host factors involved in viral entry and replication. Viral entry occurs by binding to the receptor (i.e., sialic acid) on the cell surface to induce endocytosis. Once virus is within an endosome, the host factor **(1)** PLA2G16 is recruited during pore formation and mediates viral uncoating allowing the positive sense (+) viral RNA genome to enter the cytoplasm. The viral genome associates with a replication organelle where **(2)** several host factors (i.e., Sam68, hnRNP A1, and FBP1) are recruited and bind to the IRES to promote translation of the viral genome using the cap-independent process while miR-134 binding to IRES inhibits protein translation. The generated polyprotein is cleaved into capsid proteins and replication proteins. Viral replication takes place on the membranes of replication organelles where several **(3)** host factors (i.e., ACBD3, PI4KB, SETD3, and PHB) along with viral proteins (i.e., 2A and 3A) are involved in genome replication. Capsid proteins self-assemble and genome maturation occurs which leads to viral egress using either lytic or non-lytic methods processes.

Viral entry and replication rely heavily on numerous host factors and are fundamental processes dictating the success of a viral infection (Figure 1). Many studies have identified host factors that are critical for EV propagation (reviewed in Owino and Chu, 2019). However, much of this work was performed on non-neuronal cell types which have shown little overlap between the deregulated host proteins identified during EV infected motor neurons (Too et al., 2018), reinforcing the need to verify the role of these host factors in CNS relevant models.

Once the EV invades the host cell, the viral genome needs to escape from the endosome prior to degradation in a process called uncoating. The EV genome is released from the endosome by the formation of pores within the membrane which allow the genome to be translocated into the cytoplasm (Strauss et al., 2013; Panjwani et al., 2014). This process is typically initiated in the presence of either low pH within the endosome or triggered by viral binding to a receptor. For example, poliovirus and CVB3 require binding to their receptors for uncoating while EV-A71 also needs an acidic endosome (Hussain et al., 2011). This uncoating process promotes the conversion of the viral genome into an alternate state which favors membrane interaction (Tuthill et al., 2006) and expedites viral replication and protein translation. One host factor implicated in viral uncoating is a lipid modifying enzyme called PLA2G16. The importance of this host factor was demonstrated when HeLa cells expressing enzymatically inactive PLA2G16 were resistant to EV infection and mice deficient in this gene were largely resistant to developing paralysis and succumbing to infection by CVA4 or CVA10 (Staring et al., 2017). The authors found that during viral entry, the pore-activated process which results in the degradation of the viral particles also initiates recruitment of the phospholipase PLA2G16 that allows for the release of the viral genome into the cytoplasm (Staring et al., 2017). This host factor was recently found to have pan-enteroviral properties for EVs that bind to sialic acid receptors for entry (Baggen et al., 2019). However, certain EVs using the sulfated glycosaminoglycans (sGAGs) for viral entry can be independent of PLA2G16 (Baggen et al., 2019).

As the positive-sense RNA genome enters the cytoplasm, replication primarily occurs on rearranged membranous structures termed replication organelles (ROs) that originate at the endoplasmic reticulum and then the *trans*-Golgi network as observed for CVB3 infection of Vero E6 cells (Melia et al., 2019). However, infection of motor neurons by EV-A71 showed that viral replication occurred on the mitochondrial membrane (Too et al., 2018) and further studies are warranted to identify if this is a neuronal-specific mechanism. In general, enteroviruses enrirch these membranes with both lipids and cholesterol to facilitate the formation of ROs, which are essential for viral replication (Hsu et al., 2010; van der Schaar et al., 2013; Arita, 2014; Roulin et al., 2014; Strating et al., 2015). This is mediated by the viral protein 3A indirectly recruiting host factor PI4KB (Greninger et al., 2012). In fact, the 3A proteins of several enteroviral species including EV-A, -B, -C and -D recruit the host factor ACBD3 that serves to not only recruit PI4KB but also scaffolds other viral and host proteins for proper RO formation (Lyoo et al., 2019). However, biogenesis of ROs are not essential to the initiation of viral replication since viral replication still occurred in cells when RO formation was delayed (Melia et al., 2017). Another host factor critical for viral RNA replication is the methyltransferase SET domain containing 3 (SETD3) (Diep et al., 2019). The authors showed that the cytosolic form of the actin histadine methyltransferase SETD3 interacts with the viral 2A protease from multiple enteroviral species including EV-D68, EV-A71 and CVA10 and this interaction was important for viral RNA replication (Diep et al., 2019). Animals with *Setd3*^–/–^ were completely resistant to EV viral infection when injected intracranially or intramuscularly (Diep et al., 2019), supporting the necessity of usurping these mechanisms by EVs within the CNS.

Another major phase of viral replication is viral protein translation. Several host factors are important in mediating translation of the viral polyprotein by associating with the internal ribosome entry site (IRES) located within the 5′ untranslated region (5′ UTR) of the viral genome. Mutations within the 5′ UTR were shown to modulate neurovirulence of poliovirus (De Jesus et al., 2005), EV-A71 (Yeh et al., 2011) and CVA16 (Li et al., 2016). A recent study using transcriptomic analysis showed that CVA16 but not EV-A71 infected neuroblastoma cell line SK-N-SH induced the expression of an interferon stimulated gene (ISG) called RSAD2 (Yogarajah et al., 2018). The authors found that differences within the 5’ UTR structures were the reason for this neuronal-specific modulation of RSAD2 (Yogarajah et al., 2018). Certain host proteins function as internal ribosome entry sites transacting factors (ITAFs) that help recruit ribosomes to the IRES. The heterogenous nuclear ribonucleoprotein (hnRNP) A1 was found to bind to the IRES of EV-A71 (Levengood et al., 2013) and induce a conformational change that enhanced viral protein translation (Tolbert et al., 2017). Similar positive effects by other ITAFs such as FBP2 (Huang et al., 2011) and Sam68 (Zhang et al., 2015) were observed in EV-A71 protein translation. In turn, some host factors were identified to inhibit viral protein translation. A recent study found that miR-134 binds to the IRES of Sabin 1 but not Sabin 2 poliovirus, causing degradation and modest inhibition of viral titer (Bakre et al., 2017). This miRNA binding site was conserved across other enteroviruses, such as EV-A71, indicating a universal regulation of viral replication within the host cell (Bakre et al., 2017). MiRNA expression is highly tissue-specific where miR-134 is readily expressed in the brain (Huang et al., 2015) and tissue tropism effects, such as viral inhibition or propagation, due to miRNAs were reported for numerous viruses (Orr-Burks et al., 2017). During viral infection, even modest effects may provide the necessary edge required for inhibited viral dissemination, prevention of fatal infections or providing sufficient restriction on viral replication for effective viral clearance by the immune response. However, the tropism of enteroviruses is not dictated solely by the IRES-5′UTR sequence. A study using recombinant human adenoviruses to express IRES of wild-type and Sabin 3 polioviruses as well as CVB3 revealed that infecting animals with these viral constructs resulted in viral protein translation in many organs, including sites where wild-type virus was unable to normally replicate (Kauder and Racaniello, 2004). The authors concluded that tropism of enteroviruses is likely not directly related to potential attenuation of IRES-mediated translation but rather occurs either upstream or downstream of this process (Kauder and Racaniello, 2004). Additional studies are needed to identify these tropism factors.

A recent publication identified a neuronal-specific host factor that was implicated in modulating EV-A71 neuropathogenesis in animals. The host factor prohibitin (PHB) was upregulated during EV-A71 infection of the motor neuron cell culture NSC-34 and found to contribute to not only binding of virions for cell entry but mediating viral scaffold formation on mitochondria (Too et al., 2018). Inhibiting PHB after viral infection using an anti-cancer drug Roc-A induced mitochondrial destabilization and reduced intracellular ATP which, in turn, hindered viral replication (Too et al., 2018). Inhibiting PHB in an animal model of EV-A71 neuropathogenesis caused a delay in the development of neurological symptoms, prolonged death of these animals and decreased viral load within the spinal cord and brain (Too et al., 2018), highlighting the pro-viral role of the host factor PHB in neurons.

As Enteroviruses hijack the host cell they are able to manipulate the cell cycle progression to generate a favorable cellular environment for viral replication. EV-A71 and CVA16 infected RD cells stopped cell cycle in the S phase, mediated by the non-structural 3D viral protein (Yu et al., 2015). In contrast, both EV-D68 and CVA6 induces cell cycle arrest in the G0/G1 phase due to expression of non-structural 3C and 3D viral proteins and subsequent modulation of cyclins and cyclin-dependent kinases (Wang Z. Y. et al., 2017; Wang Z. et al., 2018). Interestingly, recent data suggests that the CVB3 viral capsid protein, VP1, induces cell cycle arrest in G1 phase by increasing the expression of heat shock protein 70 (Wang Y. et al., 2019). These studies revealed the diverse preference EVs have for a particular stage of the cell cycle but the reason for this preference remains elusive. In terms of the CNS, mature neurons stay in post-mitotic senescence (G0) but can re-enter the cell cycle under certain circumstances. However, these neurons induce a cell cycle checkpoint arrest at G1/S phase and slowly die: a process called “abortive cell cycle re-entry” (Frade and Ovejero-Benito, 2015). The restrictive growth conditions within neurons likely contributes to the observed tissue tropism of EVs where EV-D68 can replicate in mature neurons but shows hindered viral growth (Rosenfeld et al., 2019) while EV-A71 was preferentially detected within astrocytes (Feng et al., 2016).

### Immunological Mechanisms Evoked by Enterovirus During CNS Infection

The immune response is an essential defense mechanism that combats viral infections through initiating innate and adaptive immune responses. Tight control over the immune response within the CNS tissue is especially critical in mitigating deleterious “bystander” effects while combating the viral infection. Contrarily, enteroviruses need to possess effective immune countermeasures to successfully reach the CNS and cause disease. Numerous studies describe the host immune response to EV infection and subsequent viral evasion mechanisms (reviewed in Lei et al., 2016; Jin et al., 2018). The majority of this work investigated innate immune response using non-CNS *in vitro* cells and evaluation of these mechanisms within the CNS tissue remains to be thoroughly explored. However, local immune cells of the CNS, such as astrocytes and microglia, were found to be important in mediating protective immune responses as a result of viral infection (Hwang and Bergmann, 2018). Here we will discuss these host-virus interactions during activation of innate and adaptive immune responses in light of their relevance within the CNS.

#### Innate Immune Response and Enterovirus Countermeasures

The innate immune response is the first line of defense against invading pathogens and numerous mechanisms are in place to detect and respond to such threats. All cells are equipped with unique proteins that function as sensors called pattern-recognition receptors (PRRs) which detect conserved parts of pathogens, termed Pathogen-Associated Molecular Patterns (PAMPs), or molecules that are released by damaged cells called Damage-Associated Molecular Patterns (DAMPs) (reviewed in Amarante-Mendes et al., 2018). There are several classes of PRRs including Toll-like receptors (TLRs), retinoic acid-inducible gene I (RIG-I)-like receptors (RLRs) and NOD-like receptors (NLRs) that are stimulated during viral infections (Akira et al., 2006). Activation of these PRRs initiates a signaling cascade that induces secretion of type I IFNs and stimulates an antiviral environment within the infected and neighboring cells (Müller et al., 1994). The importance of type I IFNs in EV infection was observed when mice pretreated with type I IFN were protected from a fatal infection by EV-A71 and CVA16 (Yang et al., 2015; Sun et al., 2016). As the immune response contains numerous redundancies to effectively combat pathogens, the virus needs to circumvent these mechanisms to establish an effective CNS infection. Uncovering these immune evasion mechanisms is the first step to identify effective strategies that could protect the CNS from EV-induced neuropathogenicity.

Once the viral genome is released from the viral particle it is detected by membrane bound TLR3 (detects dsRNA) and TLR7 (detects ssRNA) sensors (Mohanty and Deshpande, 2013). A signaling cascade is initiated, activating the interferon regulator transcription factors IRF3 and IRF7 that subsequently induce expression of type I IFNs (i.e., IFN-α, IFN-β). This signaling cascade is transmitted through TIR domain-containing adaptor inducible beta interferon (TRIF) for TLR3 and mediated by MyD88 for TLR7. Type I IFN response is both virus and cell-type specific; for example, mice deficient in TLR3-TRIP had decreased survival after CVA16 infection and TRIF-mediated immunity was found to be indispensable for preventing viral entry and replication within the nervous system (Yang et al., 2015). In contrast to CVA16 infection, the TLR3-TRIF pathway had no effect on poliovirus entry into the spinal cord or brain tissue (Oshiumi et al., 2011; Abe et al., 2012) perhaps due to the mechanism that poliovirus uses for CNS invasion. Furthermore, TLR activation varied between poliovirus strains within the same neuroblastoma cell culture. Specifically, wild-type poliovirus infection of human neuroblastoma cells (SK-N-SH) delayed the innate immune response by decreasing expression of TLR3 and MDA5 for 8 h post infection (Mohanty and Deshpande, 2013). However, infection of SK-N-SH by vaccine attenuated Sabin poliovirus strain was adequately controlled by TRL7 mediated type I IFN response (Mohanty and Deshpande, 2013). These stark differences clearly contribute to enteroviral neuropathogenicity and require further study. Other enteroviruses including CVB1, CVB5, EV-A71 and CVA16 induced type I IFNs through activation of TLR7 (Chehadeh and Alkhabbaz, 2013; Song et al., 2018). Further study of CVB3 infection revealed the upregulation of TRIM21, an intracellular protein that directs virions for degradation, which was necessary in mediating type I IFN (IFN- β) antiviral response through IRF3 (Liu et al., 2018). Viral spread to other tissues was observed in TRIM21 deficient mice, but the importance of this in terms of neuroinvasion remains unexplored (Liu et al., 2018). Enteroviruses were also found to induce an antiviral state in neighboring, non-infected cells. Induction of an antiviral immune state in non-infected cells is mediated by TLR9 detection of released DAMPS from virally infected cells. This was observed for EV-A71 where an immune response was evoked in uninfected neighboring cells (Hsiao et al., 2014) as a mechanism to prevent viral spread and tissue damage. To circumvent these immune responses, EVs can influence the cell to induce autophagy which degrades the membrane bound TLR7 sensor (Song et al., 2018). Additionally, EV-A71 and EV-D68 cleave IRF7 by the viral protein 3C^pro^ (Lei et al., 2013; Xiang et al., 2016), which is also used by CVA16, CVA6 and EV-D68 to inhibit TLR3 mediated type I IFN signaling (Rui et al., 2017). Furthermore, EV-A71 expressed 2A protease downregulates the TLR3 (Chen et al., 2018) and subverts the type I IFN immune response.

Additional dsRNA sensors called RIG-I and MDA5, a member of RLR, can also detect diverse dsRNA molecules produced during viral replication and cause the induction of type I IFN stimulated genes (Kato et al., 2006). Once dsRNA is detected, these cytoplasmic sensors undergo a conformation change allowing them to associate with other proteins, including mitochondrial-associated signaling adaptor proteins (MAVS), which are required to trigger the expression of type I IFN and other inflammatory cytokines (Chen et al., 2018). Additionally, Lys 63-linked (K63-linked) polyubiquitination is required for RIG-I and MAD5 activation (reviewed in Cao, 2016; Lang et al., 2017). Poliovirus, CVB3, EV-A71, EV-D68, CVA16 and CVA6 can disrupt this host response using several redundant and sometimes common mechanisms. For instance, poliovirus, CVB3 and EV-A71 induces proteolytic degradation of MDA5 and MAVS through the viral 2A^pro^ while RIG-I cleavage occurs via 3C^pro^ (Lei et al., 2010; Wang B. et al., 2013; Feng et al., 2014). Significantly, animal studies revealed that MDA5 was important for preventing early CVB3 replication, but was not essential for inducing type I IFNs (Hühn et al., 2010). Additionally, the 3C^pro^ of CVB3 cleaved MAVS and TRIF further suppressing type I IFN response (Mukherjee et al., 2011). For EV-A71, tissue culture studies revealed that the viral RNA-dependent RNA-polymerase (3D^pol^) and viral 3C^pro^ interacts with MDA5 and antagonizes the antiviral immune response (Kuo et al., 2013, 2019; Rui et al., 2017) in addition to inhibiting RIG-I ubiquitination by 3C^pro^ (Chen et al., 2016). These host-virus interactions were largely depended on the type of virus, cell type and genetic background of the host (Francisco et al., 2019), further underlying the adaptive capabilities of enteroviruses and the need to study these viruses within CNS-relevant cells to better understand the development of neurological disease.

The production of NF-kB is an essential antiviral host defense important for stimulating numerous proinflammatory cytokines and chemokines (reviewed in Rahman and McFadden, 2011). Primary human astrocytes infected with CVB3 or CVB4 induced the expression of NF-kB and AP-1 transcription factors, which upregulates IL-8 and MCP-1 chemokine expression (Kwon et al., 2004). As these chemokines are potent chemoattractants, they may contribute to directing neutrophils and monocytes/macrophages to the site of infection within the CNS. Similarly, infection of astrocytoma cells by EV-A71 and CVA9 induced production of VCAM-1, IL-6 and IL-8 (Zhang et al., 2013), while IL-6 and IL-8 were upregulated in EV-A71 infected mouse primary astrocytes and human glioma cells (Wang H. et al., 2019). These studies suggest that EV may evoke common proinflammtory mechanisms to signal cell mediated immunity to converge within the virally infected CNS. In fact, consistently elevated IL-6 levels were observed in an EV-A71 infection neonatal mouse model resulting in severe damage to numerous organs including the brain (Khong et al., 2012) pointing to EV infected astrocytes as contributors to CNS damage. Indeed, previous studies found elevated levels of IL-6 and IL-8 in CSF of EV-A71 positive patients with encephalitis or meningoencephalitis (Li et al., 2015; Liu et al., 2018) and expression of these cytokines may be due to viral infected astrocytes. In one study, CVB3 infection of astrocytoma cells resulted in a productive but non-cytopathic infection (Zhang et al., 2013) that could contribute to viral persistence *in vivo* within the CNS. To subvert chemokine and cytokine production, EV-A71 encoded 3C^pro^ cleaves the TAK1/TAB1/TAB2/TAB3 complex upstream of NF-kB activation (Lei et al., 2014) while the viral 2C^pro^ protein suppresses formation of the NK-kB heterodimer (Du et al., 2015). The 3C^pro^ viral protein expressed by CVA16, CVA6 and EV-D68 also abrogates production of NF-kB (Rui et al., 2017). As with other immune evasion strategies, enteroviruses are equipped with a multitude of redundant processes to inhibit immune function.

Once virus is detected by the host cell and type I IFNs are stimulated, these molecules phosphorylate STAT1 and STAT2 via the JAK-STAT pathway (Horvath, 2004). The phosphate forms of STAT1 and STAT2 interact and translocate into the cell nucleus where they bind to IFN-stimulated response elements (ISRE) and induce expression of IFN-stimulated genes (ISGs), initiating an antiviral state within the host cell (reviewed in Schindler et al., 2007). Recently, EV-A71 infected mouse primary astrocytes and human glioma cells induced expression of STAT3 which interfered with STAT1 entry into the nucleus, inhibiting the production of ISGs and thereby the type I IFN-mediated antiviral response (Wang H. et al., 2019). In non-neuronal cells, this process was inhibited via blocking nuclear entry of phosphorylated STAT1/2 by degrading the nuclear transport receptor for STAT1/2 called KPNA1 (Wang C. et al., 2017). This downregulation was mediated via caspase-3 induced degradation (Wang C. et al., 2017), which was similarly observed in EV-A71 infected primary mouse astrocytes with identification of an additional layer of type I IFN regulation. The authors found that not only did EV-A71 degrade the importin required for STAT1 translocation into the nucleus by upregulating capsease-3, but the upregulated STAT3 competed with STAT1 for KPNA1 (Wang H. et al., 2019). Furthermore, EV-A71 reduced the levels of interferon receptor 1 (IFNAR1) via the viral 2A protease activity which in turn blocked IFN-mediated phosphorylation of STAT1, STAT2, Jak1 and Tyk2 (Lu et al., 2012).

Type II IFN (IFN-γ) and Type III IFN (IFN lambda) responses are also important to combat EV infections. An initial *in vitro* study found that poliovirus titers were decreased within neuronal cultures after IFN-γ induced a modest increase in Nitric Oxide (NO) production 3 days post treatment suggesting that IFN-γ does have protective, albeit slight, antiviral effects in neurons (Komatsu et al., 1996). Similarly, protective effects of IFN-γ were observed in an animal model challenged with a lethal strain of CVB3, where expression of IFN-γ decreased viral load, viral spread and tissue destruction (Henke et al., 2003). Similarly, IFN-γ is needed to inhibit severe EV-A71 infection in mice (Wang L. C. et al., 2015). To counteract this immune response, EV-A71 encoded viral proteins 2A and 3D decreased phosphorylation of STAT1 at 2 different sites and thereby inhibited IRF1 transactivation of IFN-γ (Wang L. C. et al., 2015). Type III IFN response (IFN lambda) was recently identified to play an important antiviral role on mucosal endothelial membranes. At the intestinal mucosa, type III IFN disrupted replication of CVB3 in primary human pancreatic endocrine cells (Lind et al., 2013), hepatocytes (Lind et al., 2014) and goblet cells from primary human intestinal epithelial monolayers (Good et al., 2019). However, the 2A^pro^ viral protein encoded by CVB3 degraded TRIF and MAVS, inhibiting production of type III IFN (Lind et al., 2016). It remains to be determined if the same host-virus interactions occur at the endothelial barriers surrounding the brain and spinal cord.

#### Adaptive Immune Response

The adaptive immune response is involved in clearing EVs by generating neutralizing antibodies and cell mediated responses while building a memory to protect the host from secondary exposure. However, little is known about the cellular adaptive immune response to most EVs, particularly regarding infection of the CNS tissue. We will briefly describe the main contributors of immunity which includes neutralizing antibodies, T cells and microglia and how these responses relate to EV infection of the CNS.

Neutralizing antibodies produced by B cells during EV infection help control viremia and therefore prevent viral dissemination to other organs and protect the host from subsequent infections. This process was well documented in patients who suffer from agammaglobulinemia and cannot produce antibodies. These individuals were found to have heightened susceptibility to EV infection and CNS invasion that caused chronic neuropathies (reviewed in Misbah et al., 1992). As infants and young children do not have fully developed immune systems, the lack of neutralizing antibodies is a proposed explanation for their enhanced susceptibility to enterovirus infections and subsequent complications (Wells and Coyne, 2019). Similarly, mice deficient in B cells were unable to clear the CVB3 viral infection (Mena et al., 1999). In certain circumstances, neutralizing antibodies against CV were found to exacerbate disease in part by mediating CV infection of monocytes/macrophages and lymphocytes and thereby aiding in dissemination of the virus throughout the body (Mena et al., 1999; Jarasch et al., 2007) in a process called antibody dependent enhancement (ADE) (reviewed by Sauter and Hober, 2009). Indeed, adult mice that were inoculated twice by CVB4 showed enhanced viral load in numerous organs including the brain and spinal cord (Elmastour et al., 2017).

Cellular immunity determines the outcome of an EV infection since no difference in neutralizing antibodies was observed between mild, severe and fatal cases of HFMD (Chang et al., 2007). The T cell response is important for effective EV clearance from the host but causes damage to the CNS during invasion. To date, only a few studies investigated the adaptive immune response to EV infection. B cells, CD4+ and CD8+ T cells were detected within the CNS tissue of EV-A71 infected patient and mice (Lin et al., 2009). Although these cells were detected within the CNS, they did not cause damage to uninfected tissue but rather helped combat the infection (Lin et al., 2009). This contradicts the suspected contribution of lymphocytes detected within CSF/CNS tissue and the development of neuropathology in patients with fatal EV infections (Lum et al., 1998; Wang et al., 1999; Yan et al., 2000) warranting additional studies.

A neonatal mouse model of CVB3 infection revealed that microglia/macrophages were activated during the acute phase of infection in the CNS and were detected in the hippocampus, cortex, subventricular zones, lateral ventricles and meninges (Feuer et al., 2009). These cells were found to engulf virally infected cells within the CNS (Feuer et al., 2009). Furthermore, they can serve as antigen presenting cells that can stimulate activation of cellular immunity. However, infection by CVB3 almost completely inhibits the antigen presentation by the MHC class I pathway, effectively evading the CD8+ T cell immunity (Kemball et al., 2009). Additional studies are required to dissect the role of microglia during EV-induced CNS disease.

### Mechanisms of Neuronal Cell Death

Neuronal cell death can be triggered by viral replication or the host self-destruct mechanisms to minimize damage from viral infections. Most studies point to EV-induced cell death following either pyroptosis, apoptosis or autophagy pathways. Further work into the mechanism of cell death within CNS relevant models will help identify key pathways that EVs use to induce neuronal cell death and subsequent tissue damage.

#### Pyroptosis

Pyroptosis is a programmed cell death mechanism that is characterized by caspase-1 activation, followed by DNA breakages without laddering, cell swelling, plasma membrane rupture and release of intracellular pro-inflammatory cytokines from the cell (Adamczak et al., 2014; Cai et al., 2014). This form of cell death is evoked by intracellular pathogens, mainly bacteria, as a means of escaping the inflammatory response of the host (Fink and Cookson, 2006; Cunha and Zamboni, 2013). Pyroptosis has been observed in EV-A71 infected neuroblastoma cell lines (Yogarajah et al., 2017a; Zhu et al., 2018). The mechanism of EV induced pyroptosis was found to involve upregulation of a major inflammasome gene called AIM2, along with several AIM2-mediated pyroptosis-associated genes including caspase-1 causing a decrease in EV-A71 replication within neurons (Yogarajah et al., 2017a). Furthermore, authors confirmed increased AIM2 expression in neurons within the spinal cord and medulla from EV-A71 positive autopsy specimens (Yogarajah et al., 2017a). However, CVA16 infection of SK-N-SH cells did not induce AIM2-mediated pyroptosis but rather increased expression of radical S-adenosylmethionine containing domain 2 (RSAD2) which in turn decreased viral replication (Yogarajah et al., 2018). EVs are clearly able to trigger different mechanisms of neuronal cell death that can have profound effects on disease progression.

#### Apoptosis

Apoptosis is a non-lytic cell death pathway that is largely immunologically silent and can be used to limit viral replication with minimal activation of inflammatory responses. Apoptosis can be triggered within the cell via the intrinsic pathway as a result of cell stress or extrinsic due to external signals from other cells. Intrinsic activation of apoptosis is mediated by mitochondria releasing pro-caspase-9, which is cleaved into caspase-9 and then activates caspase-3 (Brentnall et al., 2013). Extrinsic apoptosis is induced through receptor binding of tumor necrosis factor, activating caspase-8 which then activates caspase-3 (Würstle et al., 2010). In either case, activated caspase-3 interferes with many cellular processes, including DNA repair, triggering apoptotic characteristics of nuclear condensation, cell shrinking and membrane blebbing (Chan et al., 2015). *In vitro* studies showed cleaved caspase-9 in neuroblastoma SK-N-SH and SH-SY5Y cells infected with EV-A71 and CVA-A16 (Chen T.C. et al., 2007; Chan et al., 2015; Bai et al., 2019). Apoptosis was also observed in CVB3 infected rat cortical neuron cultures (Joo et al., 2002).

#### Autophagy

he process of autophagy is a conserved pathway for intracellular degradation, typically in the context of maintaining homeostasis for a multicellular organism. Macroautophagy is one of three distinct forms of autophagy and is used to degrade cytosolic proteins, damaged or superfluous organelles and intracellular pathogens (Nikoletopoulou et al., 2015). As microautophagy is the only form associated with enterovirus infection, all following references to autophagy specifically refer to macroautophagy. It can function as part of either an innate or an adaptive immune response to limit intracellular pathogen replication, such as by robbing an invading virus of cellular machinery. Some viruses have evolved mechanisms to inhibit autophagy pathways, while others have found ways to exploit the acidic lysosomal vesicles that cells use to denature and degrade proteins (Lai et al., 2016). In the context of enterovirus infection, autophagy is the process of virus-mediated cell death (Lai et al., 2016).

Autophagy is initiated through a double-walled isolation membrane called a phagophore that, when closed into a sealed vesicle, is referred to as an autophagosome (Nikoletopoulou et al., 2015). The autophagosome sometimes also fuses with an endosome, producing an amphisome. The autophagosome or amphisome then fuses with a lysosome, resulting in an autolysosome that hydrolyzes the contents of the inner membrane. Autophagy can be initiated through a range of mechanisms including starvation, oxidative stress, hypoxia, infection and organelle damage and can be highly cargo-selective (Johansen and Lamark, 2011). Autophagy serves as a tool to address a specific threat or damage including misfolded protein aggregates, mitochondrial damage or intracellular pathogens. Within the context of virus infection, autophagy is considered an antiviral mechanism to selectively degrade virions or virus-coopted machinery in autolysosomes (Mohamud and Luo, 2019). Enteroviruses initiate infection through receptor-mediated endocytosis and subsequent escape from the endosome, amphisome or autophagosome (Mohamud and Luo, 2019). The receptors vary, as do the exact mechanisms of endocytosis, but the pathways of endocytosis and autophagy overlap through their shared use of amphisomes (Mohamud and Luo, 2019). One study found that single gene knockdown of any of several autophagy related-genes (Beclin-1, Atg12, Atg14, ATC16 or LC3) inhibited echovirus 7 viral RNA release following receptor binding but had no inhibition on CVB3 (Kim and Bergelson, 2014). While echovirus 7 internalization does not appear to rely on autophagosomes or amphisomes, broader pathways regulating membrane trafficking may be responsible for this difference (Kim and Bergelson, 2014). Notably, echovirus 7 uses clathrin-dependent endocytosis while CVB3 uses caveolin-dependent endocytosis, which can indicate why disruption of host autophagy proteins may impact viral escape from internalized vesicles (Mohamud and Luo, 2019).

The role of autophagic machinery in viral replication was evidenced by the increase in autophagosome production following infection and viral replication was inhibited when genes associated with autophagosome formation or maturation were deleted (Wong et al., 2008; Huang et al., 2009; Corona et al., 2018). However, much of this work was done using single gene deletion experiments which confounded the results as some autophagy proteins are known to have other functions that may impact viral replication through other pathways (Mohamud and Luo, 2019). For example, knockdown of autophagy proteins ATG13 and RB1CC1/FIP200 enhanced replication of EV-A71, CVA21 and CVB3, while deletion of other autophagy components that make up the ULK1/2 complex did not affect viral replication (Mauthe et al., 2016).

The exact mechanisms enteroviruses use to enhance cellular autophagy are unknown (Mohamud and Luo, 2019). The signaling cascade of starvation-induced autophagy uses AMP activated protein kinase (AMPK) and mTORC1 to activate autophagy initiating complexes, such as ULK1/2. Poliovirus-induced autophagy is independent of the ULK1 complex and mTORC1 activity and phosphorylation does not change during poliovirus infection (Corona Velazquez et al., 2018). Enteroviruses appear to employe multiple strategies to initiate autophagy independent of the traditional nutrient-sensing AMPKs. Enterovirus-infected cells show an accumulation of autophagosomes resulting from some combination of increased autophagosome synthesis and decreased autolysosome fusion (Mohamud and Luo, 2019). The rate of autophagy degradation is difficult to measure with confidence because the virus can impact host protein expression and degradation, either directly, such as through viral proteases, or indirectly by affecting host transcription and translation or disrupting degradation pathways (Mohamud and Luo, 2019).

Attempts to study autophagy flux by monitoring the cargo receptor SQSTM1/p62 have been undermined through the discovery that it is cleaved by viral protease 2A during poliovirus, EV-D68, CVB3 and human rhinovirus 1A (Corona et al., 2018). SQSTM1/p62 and CALCOCO2/NDP52 bind enterovirus capsid protein VP1 to initiate autosomal degradation of virions, the first-identified virophagy receptor (Mohamud et al., 2019). VP1 was demonstrated to undergo ubiquitination, possibly leading to ubiquitin-dependent degradation as a means of virophagy (Mohamud et al., 2019). Supporting the observation that CVB3 protease 2A cleaves SQSTM1/p62 to inhibit virophagy (Corona et al., 2018), CVB3 showed enhanced viral growth in SQSTM1/p62 knockdown HeLa cells while reduction of host CALCOCO2 inhibited viral growth (Mohamud et al., 2019). CVB3 protease 2A cleavage of SWSTM1 inhibits its ability to bind viral capsid while CALCOCO2, whether full length or cleaved by viral protease 3C, are functional at suppressing host type I IFN antiviral response by promoting autophagy of host MAVS (Mohamud et al., 2019). The role of autophagy in innate or adaptive immunity has been well described as autophagy can be triggered by PAMPs and DAMPs (Deretic et al., 2013). These responses are often activated in concert with endosomal TLRs to stimulate other responses, such as type I IFN (Deretic et al., 2013). Autophagy is also used to degrade pro-inflammatory signaling factors, which suggests that autophagy contributes to controlling inflammatory responses after pathogens are eliminated; if autophagic clearance is insufficient to address the threat then inflammation can trigger a systemic response (Deretic et al., 2013). Cleverly, enteroviruses are able to use autophagy machinery to propagate within cytoplasmic vesicles, which can conceal them from adaptive immune surveillance and be used as vehicles for non-lytic release from the cell (Corona et al., 2018). EV-D68 is able to manipulate soluble *N*-ethylmaleimide-sensitive factor attachment receptor (SNARE) proteins to inhibit autophagosome-lysosome fusion, diverting vesicles from degradative autophagy to secretory autophagy, leading to exocytosis and vesicle fusion with the plasma membrane (Corona et al., 2018).

Autophagy is a transcriptionally regulated process (Di Malta et al., 2019) and is sensitive to the transcriptional dysregulation and inhibition caused by enterovirus infection. Post-translational regulation through autophagy proteins provides finer control of these mechanisms but some of these proteins, such as SQSTM1/p62 and CALCOCO2/NDP52, have been shown to interact with enterovirus proteins or to be targeted by enterovirus proteases (Mohamud et al., 2019). Because autophagy functions as a housekeeper in neurons, there is a basal level of autophagy in healthy cells and increases or decreases in autophagy flux correlate with neurodegeneration (Lee, 2012). As a result, EV-induced dysregulation of host autophagy can have a directly deleterious effect on cells due to the loss of autophagy’s normal protective functions (Xi et al., 2013). Neurons are post-mitotic cells that are more prone to the accumulation of toxic metabolic byproducts than dividing cells, which experience higher regeneration of cytoplasm and organelles (Lee, 2012; Meng et al., 2019). Because of the high energy demands of the brain, neurons are sensitive to starvation, which is known to initiate autophagy through the mammalian target of rapamycin (mTOR) pathway (Heras-Sandoval et al., 2019). Conversely, inhibition of autophagy has been shown to cause neurodegeneration in mice (Komatsu et al., 2006) while knockout of autophagy genes in mice showed a range of neurodefective phenotypes; autophagy was also impaired in glial cells but the role of autophagy in glia and the effects of its impairment have not been explored sufficiently at this time to draw any conclusions (Nikoletopoulou et al., 2015).

## Concluding Remarks

A growing number of Enteroviruses are associated with life-threatening neurological diseases including encephalitis, meningitis and AFM. The lack of an effective treatment for non-polio enteroviruses underscores the vital need for better understanding of their neuropathogenesis.

Despite extensive studies that have uncovered many important aspects of EV infection of the CNS and resulting pathogenesis, much still remains to be learned with respect to mechanisms of EV neuroinvasion, neurotropism and molecular pathogenicity upon viral entry into the CNS. For example, neurotropic EVs can recognize a diverse range of receptors and attachment factors for cell entry, however, the location of these receptors within CNS tissue are yet to be correlated with observed pathology and clinical symptom manifestation. Although molecular mechanisms of viral-host interaction in terms of innate immunity are well documented, the extent of this interaction in CNS relevant cell-types are mainly understudied. Recent studies have implicated the immune response to be an essential component of EV neuropathogenicity, further emphasizing the need to characterize these immune-specific mechanisms. In terms of viral neurotropism, all tested neurotropic EVs were capable of infecting neurons and/or glia cells but viral propagation was found to be largely dependent on viral strain, cell type and genetics of the host. Further studies are therefore needed to delineate these parameters and identify intrinsic host factors that are key for viral entry and replication within CNS-relevant cell-types. Considering that viral life-cycle is dependent on a multitude of host factors, identifying and characterizing these essential elements will provide inroads into designing novel treatments to abrogate EV infection of the CNS.

## Author Contributions

AnM and AlM conceptualized the review, performed the literature search, and prepared the manuscript. TB provided the critical input and revisions for the manuscript.

## Conflict of Interest

The authors declare that the research was conducted in the absence of any commercial or financial relationships that could be construed as a potential conflict of interest.
